# The Role of HLA-B Typing in Behçet’s Disease and Spondyloarthritis: Genetic and Clinical Insights

**DOI:** 10.3390/life16030409

**Published:** 2026-03-03

**Authors:** Elena Bischoff, Stoyanka Vladeva, Fabian Bischoff, Nikola Kirilov

**Affiliations:** 1 Department of Health Care, Faculty of Medicine, Trakia University, 6000 Stara Zagora, Bulgaria; 2 Rheumatology Practice Stara Zagora, 6000 Stara Zagora, Bulgaria; 3 Department of Orthopedics and Traumatology, University Hospital UMBAL Dr. Georgi Stranski, Medical University of Pleven, 5803 Pleven, Bulgaria

**Keywords:** Behçet’s disease, spondyloarthritis, HLA typing, genetic susceptibility, clinical features

## Abstract

Background: Behçet’s disease (BD) is a systemic inflammatory disorder marked by recurrent mucocutaneous and ocular manifestations, predominantly affecting populations along the historic Silk Road. Genetic susceptibility, especially involving HLA-B*51, is well established. Spondyloarthritis (SpA) shares immunogenetic and clinical overlaps with BD, notably through associations with HLA class I alleles, particularly HLA-B*27. However, extended HLA-B allele profiling in these conditions remains limited. This study aimed to investigate the extended distribution of HLA-B alleles in patients presenting with clinical features suggestive of BD or SpA and to compare their clinical and laboratory profiles. Methods: In a prospective observational study at a Bulgarian rheumatology center, 120 patients with suspected BD or SpA were enrolled between January 2023 and June 2025. Diagnoses were confirmed using International Criteria for Behçet’s Disease (ICBD) and ASAS criteria for SpA. Comprehensive clinical evaluations, laboratory assessments including HLA-B typing by Sanger sequencing, and inflammatory markers were collected and analyzed. Results: Of the cohort, 15 patients (12.5%) were diagnosed with BD and 30 (25%) with SpA. HLA-B*51 was predominantly associated with BD, while HLA-B*27 and its heterozygous combinations were prevalent in SpA patients. Suspected BD patients exhibited significantly higher levels of inflammatory markers (CRP, ESR) and characteristic clinical features such as oral/genital ulcers and uveitis compared to non-BD patients. Suspected SpA patients showed longer disease duration, increased NSAID use and higher prevalence of enthesitis, psoriasis and peripheral arthritis compared to non-SpA patients. Conclusions: This study confirms the strong associations of HLA-B*51 with Behçet’s disease and HLA-B*27 with spondyloarthritis while revealing additional heterozygous and less common alleles that suggest a broader genetic influence. These findings highlight the genetic diversity and clinical heterogeneity of BD and SpA, supporting the use of extended HLA typing to improve the diagnosis and understanding of these diseases.

## 1. Introduction

Behçet’s disease (BD) is a chronic, relapsing, systemic inflammatory disorder of unknown etiology, characterized by recurrent oral and genital ulcers, skin lesions and uveitis, often with multi-organ involvement [1]. Although BD occurs globally, its prevalence is highest along the historical Silk Road—stretching from East Asia (Japan, China) through the Middle East (Iran, Turkey) to the Mediterranean. This geographic distribution has led to its designation as the “Silk Road Disease” [2].

Despite decades of clinical research, the pathogenesis of BD remains incompletely understood. Genetic factors, particularly the HLA-B*51 allele, are well-established contributors to disease susceptibility. Numerous studies have consistently demonstrated a strong association between BD and HLA-B*51, especially in populations from Turkey, Iran and Japan [3,4,5]. Other class I HLA alleles including HLA-B*15, B*27, B*57 and A*26 have also been implicated as risk factors in various populations [6,7]. Supporting the role of genetics, Sornsamdang et al. demonstrated a strong linkage disequilibrium between HLA-B*51:01:01 and HLA-C*14:02:01 in BD patients with the association remaining significant after Bonferroni correction (Pc = 0.02), suggesting a synergistic effect in disease development [8].

These findings support the classification of BD as an autoinflammatory disorder, shaped by both genetic predisposition and environmental triggers. Notably, McGonagle et al. proposed that BD, psoriasis, psoriatic arthritis (PsA) and spondyloarthritis (SpA) share overlapping immunopathogenic mechanisms [9]. All are linked to MHC class I alleles, particularly HLA-B*51, HLA-B*27 and HLA-C*06:02, although the strength and pattern of these associations differ [10]. Among these, HLA-B*27 is a well-established genetic marker for SpA and has been observed in some BD patients, suggesting possible shared pathways.

The classification of BD within the broader seronegative spondyloarthropathy spectrum dates back to the 1970s, when Moll et al. proposed grouping BD with SpA based on overlapping clinical features [11]. However, this remains controversial. Unlike SpA, sacroiliitis and axial skeletal involvement are rare in BD. Moreover, BD is predominantly associated with HLA-B*51, while SpA is more closely linked to HLA-B*27. Despite these differences, case reports have described patients with overlapping features of both BD and SpA, supporting potential clinical and genetic convergence [12,13,14].

Clinically, BD, SpA, and PsA share extra-articular manifestations such as uveitis, erythema nodosum and gastrointestinal symptoms. All respond to TNF-α inhibitors and share susceptibility loci involving IL-23R and IL-10 polymorphisms—variants also linked to inflammatory bowel disease. Additionally, the suspected role of microbial triggers in BD resembles mechanisms seen in reactive arthritis [15].

Traditionally, HLA testing has focused on detecting well-known disease-associated alleles like HLA-B*51 (BD) and HLA-B*27 (SpA). However, advances in molecular genotyping now enable high-resolution HLA typing, allowing for the detection of rare or underrecognized alleles. This broader approach can help identify novel risk or protective alleles, clarify overlapping syndromes and contribute to a more personalized understanding of inflammatory disease mechanisms [16,17].

Aims of the Study

This study investigates the distribution of HLA-B alleles in a real-world clinical cohort. Specifically, we compare allele frequencies among patients with BD, SpA and other inflammatory conditions. Additionally, we analyze and compare clinical and laboratory parameters between patients with suspected BD and without BD, as well as between those with suspected SpA and without SpA.

## 2. Material and Methods

Study Design and Participants

This prospective observational study was conducted at a Rheumatology center in Stara Zagora, Bulgaria. Between January 2023 and June 2025, a total of 120 patients presenting with clinical signs of an inflammatory rheumatic disease with clinical features suggestive of SpA or BD, pending further evaluation, were enrolled. Patients of all ages were eligible for inclusion, provided that informed consent was obtained (or assent along with guardian consent for minors). Patients were excluded if they did not provide informed consent; had a prior confirmed diagnosis of BD, SpA, or other autoimmune or autoinflammatory diseases; had an active infection or malignancy; were unable to complete study procedures; or had cognitive or language barriers that interfered with data collection.

At the baseline visit, demographic and clinical data were collected and patients underwent a preliminary evaluation based on symptoms and signs. Only patients whose clinical presentation was consistent with a possible diagnosis of SpA or BD were included. Patients whose features did not align with either condition were excluded. An additional follow-up visit was conducted to complete the diagnostic work-up, during which, further laboratory tests, imaging studies and specialist consultations were reviewed. Final diagnoses were established based on the International Criteria for Behçet’s Disease [18] and the Assessment of SpondyloArthritis International Society [19] criteria for SpA. Participants were analyzed in two primary groups. The first group focused on BD and was subdivided into patients with confirmed diagnoses of BD and patients without BD. The second group focused on SpA and was subdivided into patients with confirmed diagnoses of SpA and patients without SpA [20,21].

Clinical and Laboratory Data Collection

Clinical Assessments

All participants underwent a comprehensive clinical evaluation including detailed medical history and physical examination. Clinical data collected included demographic information (age, sex), body mass index (BMI), duration of low back pain and morning stiffness as well presence of characteristic features associated with SpA and BD such as peripheral arthritis, enthesitis, psoriasis, uveitis, fatigue, oral and genital ulcers, skin lesions, vascular involvement and neurological symptoms.

Laboratory Assessments

Venous blood samples were collected from all participants following standard protocols. The HLA-B locus was typed to identify allelic variants. As HLA-B testing in this clinical context serves as a diagnostic adjunct to support disease classification rather than predictive genetic screening, formal pre-test genetic counseling was not routinely conducted. All patients were informed about the purpose, scope and potential implications of HLA testing as part of the standard informed consent process for diagnostic procedures.

Inflammatory markers were measured, including C-reactive protein (CRP) levels (mg/L) and erythrocyte sedimentation rate (ESR) (mm/h). Complete blood counts were performed to assess hemoglobin concentration (HGB; g/L), red blood cell count (RBC; ×10^12^/L), hematocrit (HCT; L/L), mean corpuscular volume (MCV; fL), mean corpuscular hemoglobin (MCH; pg), mean corpuscular hemoglobin concentration (MCHC; g/L), platelet count (PLT; ×10^9^/L) and white blood cell count (WBC; ×10^9^/L).

Statistical Analysis

Statistical comparisons between patients with suspected BD and without BD and patients with suspected SpA and without SpA were performed using SPSS v21 (IBM Corp., Armonk, NY, USA). Continuous variables were summarized as means, standard deviations (SDs) and standard error of the mean (SEMs). Group comparisons were made using independent sample t-tests for continuous data and chi-square (χ^2^) tests for categorical variables. A *p*-value of <0.05 was considered statistically significant.

## 3. Results

**Figure 1:**
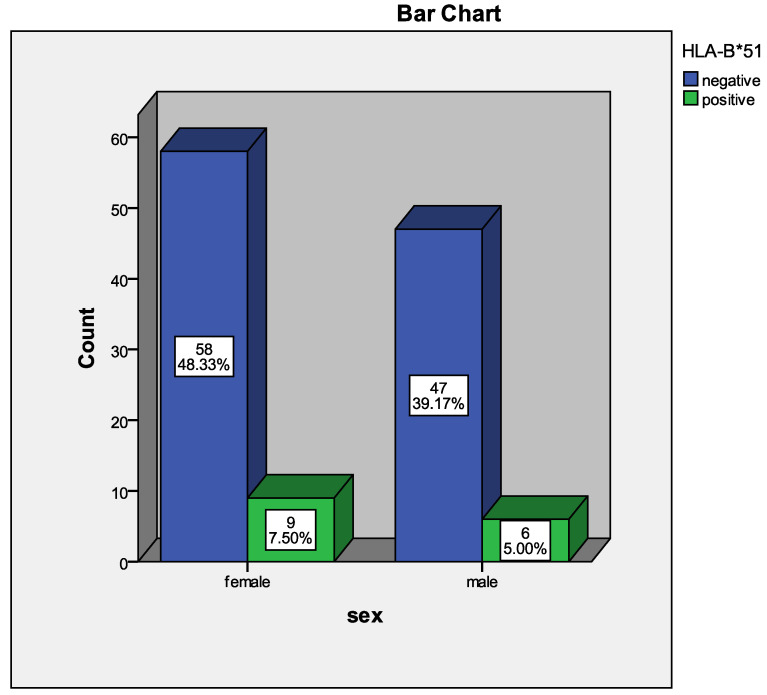
Sex-based distribution of HLA-B*51 positivity among study participants.

A total of 120 patients were included in the cohort, with a mean age of 50 years (range: 15–75 years). The mean BMI for all 120 patients was 26.1 ± 4.0 kg/m^2^. HLA-B allele typing was performed for all participants. In the total cohort, 55.8% (n = 67) were female and 44.2% (n = 53) were male. A total of 15 patients (12.5%) had a confirmed diagnosis of Behçet’s disease (BD), all of whom were HLA-B*51 positive, as shown in Figure 1. A diagnosis of SpA was established in 30 patients (25%), while the remaining 75 patients (62.5%) presented with SpA-like symptoms but did not meet the diagnostic criteria.

A total of 120 patients exhibited diverse HLA-B heterozygous genotypes with the most frequent combination being HLA-B*15/B*27, which was observed in 7 individuals (5.8%). Other relatively common combinations included B*27/B*35, B*27/B*55 and B*18/B*35. Several combinations such as B*13/B*18, B*14/B*40 and B*18/B*38 were detected in 3–4 patients each. Notably, associated heterozygous combinations involving HLA-B*51 and other HLA-B alleles (including B*07/B*51, B*08/B*51, B*13/B*51, B*15/B*51, B*18/B*51, B*35/B*51, B*37/B*51, B*40/B*51, B*44/B*51, B*51/B*52, B*51/B*56 and B*51/B*57) were detected in a total of 15 individuals diagnosed with BD. The most common combination among BD patients was HLA-B*18/B*51.

Similarly, HLA-B*27 and its associated heterozygous combinations (including B*13/B*27, B*15/B*27, B*27/B*35, B*27/B*49 and B*27/B*55) were identified in 25 individuals with SpA. The most frequently observed heterozygous combination among SpA patients was HLA-B*15/B*27 (Table 1).

Description of clinical and laboratory parameters in patients with suspected BD and without BD

The study included 120 patients, of whom, 20 were classified as having suspected BD and 85 served as the comparative group without a BD diagnosis. Among patients with suspected BD, 35% were female (7/20) and 65% were male (13/20) compared to 60% female (51/85) and 40% male (34/85) in the group without BD. The mean BMI was 26.0 ± 3.2 kg/m^2^ in the suspected BD group and 25.5 ± 4.0 kg/m^2^ in the non-BD group.

Significant differences were observed between the groups in both clinical and laboratory parameters. Patients with suspected BD were younger than those without BD (mean age: 45 ± 12 vs. 52 ± 12.5 years; *p* = 0.02). Markers of systemic inflammation, including CRP and ESR, were higher in the suspected BD group compared to the comparative group (CRP: 15.0 ± 12.0 mg/L vs. 6.5 ± 12.0 mg/L, *p* = 0.03; ESR: 28.0 ± 10.0 mm/h vs. 17.0 ± 8.0 mm/h, *p* = 0.04). PLT and WBC counts were also elevated in patients with suspected BD (PLT: 280.0 ± 80.0 × 10^3^/μL vs. 250.0 ± 70.0 × 10^3^/μL, *p* = 0.05; WBC: 8.5 ± 2.0 × 10^3^/μL vs. 7.0 ± 1.8 × 10^3^/μL, *p* = 0.04).

No statistically significant differences were observed in RBC, HGB, MCV, MCH, MCHC or HCT, although these parameters tended to be slightly higher in the suspected BD group.

Clinically, characteristic Behçet’s manifestations were more prevalent in the suspected BD group compared to the non-BD group: oral ulcers (60% vs. 4%; *p* = 0.01), genital ulcers (40% vs. 2%; *p* = 0.02), skin lesions (50% vs. 3%; *p* = 0.02), uveitis (35% vs. 2%; *p* = 0.03), joint involvement (25% vs. 6%; *p* = 0.04), vascular involvement (20% vs. 1%; *p* = 0.04), and neurological symptoms (15% vs. 1%; *p* = 0.05), Table 2.

Comparison of clinical and laboratory parameters in patients with suspected SpA and without SpA

Among the 90 patients analyzed after excluding confirmed SpA cases, 40 were classified as suspected SpA and 50 served as the comparative group without SpA. In the suspected SpA group, 38% were female (15/40) and 62% were male (25/40) compared to 60% female (30/50) and 40% male (20/50) in the group without SpA. The mean BMI was 26.5 ± 3.5 kg/m^2^ in the suspected SpA group and 27.0 ± 4.0 kg/m^2^ in the non-SpA group. This difference was not statistically significant.

Patients with suspected SpA were younger than those without SpA (mean age: 45 ± 11 vs. 53 ± 12 years; *p* = 0.03). Markers of systemic inflammation, including CRP and ESR, were higher in the suspected SpA group compared to the comparative group (CRP: 14.0 ± 12.0 mg/L vs. 6.0 ± 10.0 mg/L, *p* = 0.04; ESR: 12.0 ± 7.0 mm/h vs. 8.5 ± 5.0 mm/h, *p* = 0.05). Hemoglobin levels were slightly lower in the suspected SpA group (140.0 ± 13 g/L vs. 145.0 ± 14 g/L; *p* = 0.10), but the difference was not statistically significant.

No statistically significant differences were found for RBC, MCV, MCH, MCHC, HCT, PLT, WBC or BMI.

Clinically, patients with suspected SpA exhibited longer morning stiffness (30 ± 12 min vs. 16 ± 6 min; *p* = 0.02) and longer duration of back pain (7.0 ± 3.0 years vs. 2.0 ± 1.0 years; *p* = 0.03). HLA-B27 positivity was markedly higher among suspected SpA patients (55% vs. 6%; *p* = 0.001).

Furthermore, several SpA-related clinical features were more prevalent in the suspected SpA group compared to those without SpA: uveitis (25% vs. 3%; *p* = 0.02), enthesitis (50% vs. 7%; *p* = 0.03), psoriasis (20% vs. 4%; *p* = 0.04), fatigue (50% vs. 20%; *p* = 0.04) and peripheral arthritis (40% vs. 8%; *p* = 0.03) (Table 3).

Comparison of clinical and laboratory parameters between confirmed BD and confirmed SpA patients

Among the confirmed cases, 15 patients had BD and 30 patients had SpA. The mean age was similar between groups (BD: 41 ± 11 years; SpA: 42 ± 11 years; *p* = 0.65), and BMI did not differ significantly (BD: 25.8 ± 3.4 kg/m^2^; SpA: 26.1 ± 3.7 kg/m^2^; *p* = 0.70).

Laboratory markers of systemic inflammation showed distinct patterns between the two conditions. ESR was significantly higher in BD patients compared to SpA patients (34.1 ± 12.7 mm/h vs. 14.6 ± 7.3 mm/h; *p* = 0.01), while CRP and WBC levels did not differ significantly. Hemoglobin levels were higher in BD patients (154.8 ± 11.4 g/L) compared to SpA patients (134.7 ± 13.2 g/L; *p* = 0.002). Platelet counts were slightly elevated in BD patients (312.5 ± 84.3 × 10^3^/μL vs. 290 ± 96 × 10^3^/μL), but this difference was not statistically significant.

Clinically, BD patients exhibited markedly higher rates of mucocutaneous involvement, including oral ulcers (87% vs. 5%; *p* < 0.001), genital ulcers (53% vs. 2%; *p* < 0.001) and skin lesions (60% vs. 4%; *p* < 0.001). Uveitis was more frequent in BD than in SpA (40% vs. 28%), though this difference did not reach statistical significance (*p* = 0.30). Conversely, SpA patients showed a higher prevalence of enthesitis (56% vs. 27%; *p* = 0.02), while vascular involvement (27% vs. 10%; *p* = 0.05) and neurological symptoms (20% vs. 5%; *p* = 0.03) were more common in BD patients. Joint involvement was comparable between groups (33% (BD) vs. 42% (SpA); *p* = 0.50).

These findings highlight that confirmed BD is primarily characterized by mucocutaneous, vascular and neurological features, whereas confirmed SpA shows greater musculoskeletal involvement, particularly enthesitis. The laboratory profile also reflects these differences with higher ESR and hemoglobin in BD, helping to distinguish between the two conditions (Table 4).

## 4. Discussion

Genetic diversity of HLA-B alleles in BD

This study provides a detailed analysis of HLA-B allele diversity in patients diagnosed with BD, SpA and those with SpA-like symptoms who do not meet diagnostic criteria. Our findings confirm the well-known associations of HLA-B*51 with BD and HLA-B*27 with SpA and also reveal a broader spectrum of less common HLA-B alleles. This highlights the genetic heterogeneity underlying these diseases and suggests that additional alleles contribute to susceptibility and disease phenotype.

The consistent detection of various heterozygous combinations involving HLA-B*51 (including B*07/B*51, B*08/B*51, B*13/B*51, B*15/B*51, B*18/B*51, B*35/B*51, B*37/B*51, B*40/B*51, B*44/B*51, B*51/B*52, B*51/B*56 and B*51/B*57) in the BD patients in our study supports its central role in disease pathogenesis. These combinations may modify immune responses, potentially driving the vasculitis and mucocutaneous symptoms characteristic of BD. The presence of diverse HLA-B*51 allele combinations suggests functional differences in antigen presentation or immune regulation that could influence disease severity or progression.

Previous research supports these observations. For example, Xavier et al. reported a strong association between HLA-B*51 and BD (*p* = 4.11 × 10^−41^; OR = 4.63) and identified HLA-B*15 as a secondary risk allele [22]. Other studies, such as those conducted in Israeli patients, have shown increased frequencies of HLA-B*51 and HLA-B*52 among BD cases, underscoring their role in familial clustering and heritable risk [23]. Interestingly, alleles like HLA-B*07 may have entered the human genome through Neanderthal introgression, possibly influencing susceptibility to autoimmune conditions like BD [24].

Beyond HLA-B51, other alleles such as B*08, B*13, B*18 and B*35 have been implicated in BD risk, reflecting the complex genetic architecture of the disease. For instance, Pekiner et al. found a higher frequency of HLA-B*13 in patients with recurrent aphthous ulcers compared to BD, indicating potential allele-specific differences in mucosal immune responses. Rare alleles like HLA-B*37 and B*40 have also been linked to familial clustering and early onset in pediatric cases, suggesting that non-classical alleles may contribute to disease heterogeneity [25,26,27,28,29,30].

HLA-B alleles and SpA

In our SpA cohort HLA-B*27 and its heterozygous combinations (such as B*13/B*27 and B*15/B*27) were prevalent. The association of HLA-B*27 with axial SpA is well documented, but its frequency varies by ethnicity and a substantial number of SpA patients do not carry this allele. In HLA-B*27-negative patients other alleles such as HLA-B*13, B*35, HLA-B*49, as well as A*29, B*38 and B*52, may play a role in disease susceptibility [31,32].

Allele-specific patterns have also been observed: HLA-B*15 is often linked to peripheral SpA, whereas HLA-B*27 is more commonly associated with axial disease. This supports the idea that different HLA-B alleles influence distinct clinical presentations within the SpA spectrum [33,34]. Moreover, the presence of HLA-B*55 in combination with B*27 in some patients may reflect shared immunogenetic mechanisms between SpA and other autoinflammatory conditions like familial Mediterranean fever [35].

Other alleles like HLA-B*40 and B*44 have also been implicated in ankylosing spondylitis. For example, HLA-B*40 is enriched among B*27-positive AS patients and may act as a disease modifier. Similarly, B*44 homozygosity or B*27/B*44 heterozygosity might increase arthritis risk, emphasizing the importance of allele interactions in disease expression [33].

It is important to note that while HLA-B typing can support diagnosis and risk stratification, knowledge of a specific HLA-B allele does not currently alter therapeutic management. Treatment decisions remain guided by clinical phenotype, disease activity, organ involvement and established therapeutic guidelines. Nevertheless, identifying HLA-associated patterns may provide prognostic information and assist clinicians in anticipating organ-specific complications, particularly in BD where HLA-B51 has been linked to uveitis and more severe disease phenotypes.

Clinical and laboratory correlations

Patients with BD showed significantly higher levels of inflammatory markers CRP and ESR, consistent with previous studies linking these markers to active disease and clinical manifestations. Elevated platelet and white blood cell counts further reflect the systemic inflammation characteristic of BD. Clinically, BD patients had significantly higher rates of oral ulcers, genital ulcers, skin lesions, uveitis, joint involvement, vascular manifestations and neurological symptoms compared to controls, although the overall prevalence was slightly lower than in some prior studies, possibly due to differences in population or disease stage [36,37,38,39,40].

Similarly, SpA patients in our study exhibited longer morning stiffness and back pain duration, along with increased HLA-B*27 positivity and higher frequencies of uveitis, enthesitis, psoriasis, fatigue and peripheral arthritis. Inflammatory markers were elevated, while hemoglobin levels were lower, indicating ongoing systemic inflammation. These findings align with previous reports describing the clinical and laboratory profiles of SpA patients [41,42,43].

Strengths and Limitations

This study’s prospective design minimizes recall bias and allows for real-time data collection. The use of standardized diagnostic criteria improves diagnostic accuracy and comparability. Comprehensive clinical and laboratory evaluations, including HLA-B typing, enable robust genotype-phenotype analyses. Follow-up visits ensured diagnostic confirmation, enhancing data validity.

However, the study is limited by its sample size and the population specificity, which may affect the generalizability of the findings. Further research in larger, ethnically diverse cohorts is needed to confirm these associations and elucidate the functional implications of less common HLA-B alleles.

## 5. Conclusions

This study provides a detailed analysis of HLA-B allele distribution in patients with BD and SpA, highlighting both well-established and less frequent genetic associations. The strong correlation between HLA-B*51 and BD, as well as HLA-B*27 and SpA, was reaffirmed while additional heterozygous combinations and non-classical alleles suggest a broader genetic contribution to disease susceptibility and phenotype. The identification of diverse HLA-B profiles—particularly in patients not meeting full diagnostic criteria—underscores the heterogeneity of these conditions and supports the value of HLA typing in clinical evaluation. These findings may aid in refining diagnostic approaches and improving our understanding of the immunogenetic underpinnings of BD and SpA. Further multicenter studies with larger, ethnically diverse cohorts and longitudinal follow-up are needed to validate and expand upon these results.

## Figures and Tables

**Table 1:** Distribution of HLA-B heterozygous allelic combinations among 120 patients.

HLA-B Genotype Combination	Number of Patients	% of 120 Subjects
B*07/B*18	2	1.7%
B*07/B*35	3	2.5%
B*07/B*44	2	1.7%
B*07/B*51	1	0.8%
B*08/B*18	3	2.5%
B*08/B*35	4	3.3%
B*08/B*40	2	1.7%
B*08/B*51	2	1.7%
B*08/B*55	2	1.7%
B*13/B*18	4	3.3%
B*13/B*27	2	1.7%
B*13/B*35	1	0.8%
B*13/B*51	1	0.8%
B*14/B*40	4	3.3%
B*14/B*50	1	0.8%
B*15/B*18	1	0.8%
B*15/B*27	7	5.8%
B*15/B*39	1	0.8%
B*15/B*49	2	1.7%
B*15/B*51	1	0.8%
B*15/B*52	2	1.7%
B*18/B*35	5	4.2%
B*18/B*38	4	3.3%
B*18/B*40	1	0.8%
B*18/B*41	2	1.7%
B*18/B*51	2	1.7%
B*18/B*55	2	1.7%
B*27/B*35	6	5.0%
B*27/B*49	5	4.2%
B*27/B*55	6	5.0%
B*35/B*39	2	1.7%
B*35/B*44	2	1.7%
B*35/B*51	1	0.8%
B*37/B*38	3	2.5%
B*37/B*51	1	0.8%
B*38/B*39	2	1.7%
B*38/B*40	3	2.5%
B*38/B*44	2	1.7%
B*39/B*44	3	2.5%
B*40/B*44	2	1.7%
B*40/B*51	1	0.8%
B*40/B*57	3	2.5%
B*41/B*44	2	1.7%
B*44/B*51	1	0.8%
B*44/B*57	3	2.5%
B*49/B*44	2	1.7%
B*51/B*52	2	1.7%
B*51/B*56	1	0.8%
B*51/B*57	1	0.8%

* The asterisk indicates allele notation.

**Table 2:** Clinical and laboratory parameters in patients with suspected BD and without BD.

Parameter	Group	N	Mean/%	SD	SEM	*p*-Value
Age (years)	Without BD	85	52	12.5	1.36	0.02
	Suspected BD	20	45	12.0	2.68	
CRP (mg/L)	Without BD	85	6.5	12.0	1.30	0.03
	Suspected BD	20	15.0	12.0	2.68	
ESR (mm/h)	Without BD	85	17.0	8.0	0.87	0.04
	Suspected BD	20	28.0	10.0	2.24	
PLT (10^3^/μL)	Without BD	85	250.0	70.0	7.59	0.05
	Suspected BD	20	280.0	80.0	17.9	
WBC (10^3^/μL)	Without BD	85	7.0	1.8	0.20	0.04
	Suspected BD	20	8.5	2.0	0.45	
RBC (10^6^/μL)	Without BD	85	4.7	0.42	0.045	0.15
	Suspected BD	20	4.85	0.45	0.10	
HGB (g/L)	Without BD	85	140.0	12.0	1.30	0.08
	Suspected BD	20	145.0	12.0	2.68	
MCV (fL)	Without BD	85	89.0	4.5	0.49	0.10
	Suspected BD	20	91.0	4.0	0.89	
MCH (pg)	Without BD	85	29.5	1.7	0.18	0.10
	Suspected BD	20	30.5	1.5	0.34	
MCHC (g/L)	Without BD	85	331.0	8.5	0.92	0.15
	Suspected BD	20	335.0	8.0	1.79	
HCT (L/L)	Without BD	85	0.420	0.030	0.0033	0.12
	Suspected BD	20	0.440	0.035	0.0078	
Oral ulcers (%)	Without BD	85	4	-	-	0.01
	Suspected BD	20	60	-	-	
Genital ulcers (%)	Without BD	85	2	-	-	0.02
	Suspected BD	20	40	-	-	
Skin lesions (%)	Without BD	85	3	-	-	0.02
	Suspected BD	20	50	-	-	
Uveitis (%)	Without BD	85	2	-	-	0.03
	Suspected BD	20	35	-	-	
Enthesitis (%)	Without BD	6	6	-	-	0.05
	Suspected BD	4	20	-	-	
Joint involvement (%)	Without BD	85	6	-	-	0.04
	Suspected BD	20	25	-	-	
Vascular involvement (%)	Without BD	85	1	-	-	0.04
	Suspected BD	20	20	-	-	
Neurological symptoms (%)	Without BD	85	1	-	-	0.05
	Suspected BD	20	15	-	-	

**Table 3:** Clinical and laboratory parameters in patients with suspected SpA and without SpA.

Variable	Group	N	Mean/%	SD	SEM	*p*-Value
Age (years)	Without SpA	50	53	12	1.70	0.03
	Suspected SpA	40	45	11	1.74	
CRP (mg/L)	Without SpA	50	6.0	10	1.41	0.04
	Suspected SpA	40	14.0	12	1.90	
ESR (mm/h)	Without SpA	50	8.5	5.0	0.71	0.05
	Suspected SpA	40	12.0	7.0	1.11	
Hemoglobin (g/L)	Without SpA	50	145.0	14	1.98	0.10
	Suspected SpA	40	140.0	13	2.05	
RBC (×10^12^/L)	Without SpA	50	4.70	0.45	0.064	0.20
	Suspected SpA	40	4.80	0.46	0.073	
MCV (fL)	Without SpA	50	91.0	5.0	0.71	0.12
	Suspected SpA	40	90.0	5.5	0.87	
MCH (pg)	Without SpA	50	30.5	2.0	0.28	0.15
	Suspected SpA	40	30.0	2.5	0.40	
MCHC (g/L)	Without SpA	50	334.0	10	1.41	0.20
	Suspected SpA	40	335.0	8	1.26	
HCT (L/L)	Without SpA	50	0.430	0.040	0.0057	0.18
	Suspected SpA	40	0.425	0.030	0.0047	
PLT (×10^9^/L)	Without SpA	50	260	85	12.0	0.10
	Suspected SpA	40	280	90	14.2	
WBC (×10^9^/L)	Without SpA	50	7.2	2.0	0.28	0.12
	Suspected SpA	40	8.5	2.0	0.32	
BMI	Without SpA	50	27.0	4.0	0.57	0.15
	Suspected SpA	40	26.5	3.5	0.55	
Morning Stiffness (min)	Without SpA	50	16	6	0.85	0.02
	Suspected SpA	40	30	12	1.90	
HLA-B27 Positivity (%)	Without SpA	50	6	-	-	0.001
	Suspected SpA	40	55	-	-	
Uveitis (%)	Without SpA	50	3	-	-	0.02
	Suspected SpA	40	25	-	-	
Enthesitis (%)	Without SpA	50	7	-	-	0.03
	Suspected SpA	40	50	-	-	
Psoriasis (%)	Without SpA	50	4	-	-	0.04
	Suspected SpA	40	20	-	-	
Back pain duration (yrs)	Without SpA	50	2.0	1.0	0.14	0.03
	Suspected SpA	40	7.0	3.0	0.47	
Fatigue (%)	Without SpA	50	20	-	-	0.04
	Suspected SpA	40	50	-	-	
Peripheral arthritis (%)	Without SpA	50	8	-	-	0.03
	Suspected SpA	40	40	-	-	

**Table 4:** Comparison of clinical and laboratory parameters of confirmed BD and confirmed SpA patients.

Parameter	BD (n = 15)	SpA (n = 30)	*p*-Value
Age (years)	41 ± 11	42 ± 11	0.65
BMI (kg/m^2^)	25.8 ± 3.4	26.1 ± 3.7	0.70
CRP (mg/L)	22.7 ± 10.5	18.7 ± 24.5	0.35
ESR (mm/h)	34.1 ± 12.7	14.6 ± 7.3	0.01
Hemoglobin (g/L)	154.8 ± 11.4	134.7 ± 13.2	0.002
WBC (×10^3^/μL)	9.6 ± 2.1	9.03 ± 1.8	0.45
PLT (×10^3^/μL)	312.5 ± 84.3	290 ± 96	0.35
Oral ulcers (%)	87	5	<0.001
Genital ulcers (%)	53	2	<0.001
Skin lesions (%)	60	4	<0.001
Uveitis (%)	40	28	0.30
Joint involvement (%)	33	42	0.50
Enthesitis (%)	27	56	0.02
Vascular involvement (%)	27	10	0.05
Neurological symptoms (%)	20	5	0.03

## Data Availability

The authors confirm that the data supporting the findings of this study are not publicly available due to privacy and ethical restrictions.
